# Exploring Determinants of Handwashing with Soap in Indonesia: A Quantitative Analysis

**DOI:** 10.3390/ijerph13090868

**Published:** 2016-09-01

**Authors:** Mitsuaki Hirai, Jay P. Graham, Kay D. Mattson, Andrea Kelsey, Supriya Mukherji, Aidan A. Cronin

**Affiliations:** 1 Milken Institute School of Public Health at the George Washington University, Washington, DC 20052, USA; mhirai@gwu.edu (M.H.); jgraham@gwu.edu (J.P.G.); kelseyak@gwmail.gwu.edu (A.K.); 2 Independent Consultant, Albany, OR 97321, USA; kdmattson11@outlook.com; 3 United Nations Children’s Fund (UNICEF), Jakarta 12920, Indonesia; supriyamukherji@gmail.com

**Keywords:** handwashing, hygiene, Indonesia, WASH, open defecation

## Abstract

Handwashing with soap is recognized as a cost-effective intervention to reduce morbidity and mortality associated with enteric and respiratory infections. This study analyzes rural Indonesian households’ hygiene behaviors and attitudes to examine how motivations for handwashing, locations of handwashing space in the household, and handwashing moments are associated with handwashing with soap as potential determinants of the behavior. The analysis was conducted using results from a UNICEF cross-sectional study of 1700 households in six districts across three provinces of Indonesia. A composite measure of handwashing with soap was developed that included self-reported handwashing, a handwashing demonstration, and observed handwashing materials and location of facilities in the home. Prevalence ratios were calculated to analyze associations between handwashing with soap and hypothesized determinants of the behavior. Our results showed that determinants that had a significant association with handwashing with soap included: (1) a desire to smell nice; (2) interpersonal influences; (3) the presence of handwashing places within 10 paces of the kitchen and the toilet; and (4) key handwashing moments when hands felt dirty, including after eating and after cleaning child stools. This study concludes that handwashing with soap may be more effectively promoted through the use of non-health messages.

## 1. Introduction

Handwashing with soap at critical moments, such as before eating and after defecation, can prevent infectious diseases by interrupting the transmission of infectious agents. Empirical evidence suggests that handwashing with soap reduces the risk of diarrhea [1,2,3], acute lower respiratory infections [4], and soil-transmitted helminths [5], and it has been recognized as one of the most cost-effective health interventions to reduce the burden of disease [6]. Yet, only 19% of the global population is estimated to wash their hands with soap after using a sanitation facility or handling children’s excreta [7].

Good hygiene is of vital importance in Indonesia due to the lack of basic sanitation in the country. Approximately 20% of the Indonesian population, or 51 million people, are estimated to defecate in open spaces, such as fields, bushes, and beaches [8]. Open defecation increases the risk of physical contact with fecal pathogens and can induce both short-term and long-term health consequences including diarrhea, stunting, and environmental enteropathy [9,10]. In this context, handwashing with soap can serve as a primary barrier to disease transmission.

The Government of Indonesia (GoI) has promoted handwashing with soap as part of the national sanitation program, Sanitasi Total Berbasis Masyarakat (STBM), or the Community Approaches to Total Sanitation and Hygiene [11]. The STBM aims to eliminate open defecation from 20,000 villages by 2019, and the five project pillars include eliminating open defecation, increasing the practice of handwashing with soap and household water treatment, and improving solid waste and wastewater management [11]. In November 2012, the United Nations Children’s Fund (UNICEF) and the GoI launched a four-year sanitation and hygiene project in the eastern provinces of Indonesia to scale up and strengthen the efforts underway in the STBM program.

Although handwashing may be understood as a simple task, multiple levels of influence or behavioral determinants exist to shape this behavior. Previous research has suggested that adult caregivers, female sex, higher levels of education, high socioeconomic status, place of residence, and access to water and sanitation are associated with handwashing with soap [4,12,13]. Habits of washing hands and motivations for cleanliness have also been identified as psychological determinants of handwashing with soap [14]. A review of formative studies from 11 countries in Asia, Africa, and South America further highlighted the roles of emotional drivers, such as disgust, comfort, and desire for social status, and suggested that health-related motivations may not always be the key determinant of handwashing behaviors [15]. Those socio-demographic, structural, and psychosocial factors identified in empirical research have informed the recent development of conceptual models and theoretical frameworks to design effective hygiene interventions [16,17,18].

Accurate measurement of behaviors is foundational for estimating the prevalence of handwashing with soap and assessing hygiene interventions. Yet, the difficulty of measuring handwashing behaviors is well-recognized [19]. Empirical evidence of handwashing practice has been collected by a number of measures including self-reports, rapid observations, microbiological assessment of hands, structured observations, and soap-sensor-based methods with varying levels of validity, feasibility, efficiency, and affordability [20]. While self-reported handwashing behaviors can be efficiently measured at relatively affordable cost, people tend to overestimate their handwashing practice [21,22]. Observing a practical handwashing demonstration is another relatively efficient approach to assess people’s handwashing practice, but limited evidence exists to determine the effectiveness of this measurement [20]. Rapid observations of handwashing resources in the household, such as presence of soap, water and a dedicated handwashing location, may provide a reliable estimate of handwashing behaviors, but they cannot assess the frequency and consistency of handwashing behaviors at critical moments for each person [20].

Structured observations, where researchers directly observe people’s handwashing behaviors for a few hours or more, can collect rich contextual data and objective measures of handwashing practice at critical moments including after defecation [23]. By assuming that structured observations can yield the best available estimate of actual handwashing behaviors, this method has been used as a standard of comparison to assess the performance of other proxy measures, such as self-reports and rapid observations [21,22,24]. This measurement, however, is often time-consuming and labor-intensive, which may not be feasible or appropriate for a large household survey. Thus, a single universal method of handwashing measurement that suits every research setting does not exist [20].

While formative research has explored how people may be motivated to wash their hands with soap in Indonesia [25,26], quantitative evidence remains limited to inform the behavioral determinants of handwashing with soap in Indonesia and associated interventions. The limitations of self-reports, handwashing demonstration, and rapid observations of handwashing resources have been identified [20], but few studies have examined the effectiveness of a composite measure to assess handwashing with soap through a household survey.

In this study, we aim to identify the potential determinants of handwashing with soap, estimated by the use of a composite measure for each household enrolled that includes: (1) self-reported handwashing with soap; (2) an observed handwashing demonstration; and (3) observation of handwashing materials (i.e., soap and water) available to household members. Applying this composite measure, the study aims to assess how motivational drivers, key times that individuals wash their hands, and the presence of a handwashing place in the household are independently, as well as collectively, associated with handwashing with soap. Respondent’s age, sex, education, place of residence, household wealth levels, access to water for household needs, and ownership of a private toilet are included in the analysis. Motivations are included as the psychological determinants of handwashing behaviors. Handwashing moments (e.g., after defecation) and the presence of a handwashing place in the household serve as physical cues to induce handwashing with soap. It is hypothesized that motivations for handwashing, self-reported handwashing moments, and access to handwashing facilities will be significantly associated with handwashing with soap, which may be collectively moderated by socio-demographic characteristics and structural factors.

## 2. Materials and Methods

In February 2014, UNICEF commissioned Taylor Nelson Sofres (TNS) Indonesia to conduct a knowledge, attitude, and practice (KAP) survey to establish a baseline of sanitation and hygiene conditions in selected communities at the beginning of STBM implementation. UNICEF specifically selected six districts, which are typically more resource-constrained and have low levels of access to sanitation in Indonesia. Based on the multi-stage cluster sampling method, 2036 households were initially identified and contacted by house-to-house visits. A total of 1786 households had someone at home, and 1700 agreed to participate in the survey, attaining the participation rate of 95.2%. The questionnaire was orally administered in the local language after obtaining verbal informed consent with TNS following international ethical standards (ESOMAR) adherence and having approval from the Government of Indonesia to undertake such surveys in Indonesia. Study participants were from six districts of Indonesia across three provinces, including Alor, Sumba Timur (NTT Province), Luwu Utara, Takalar, Barru (South Sulawesi Province), and Jayapura (Papua Province) (Table 1).

The outcome variable of this study is handwashing with soap, measured using three indicators of handwashing. First, respondents were asked what they usually use to wash their hands without any prompt. Second, the availability of water and soap at a handwashing place in the household was assessed through direct observation. Lastly, the research team asked respondents to show how they usually wash their hands and directly observed the materials used for their handwashing demonstration. Study participants were regarded as performing handwashing with soap in this study if they reported to wash hands with water and soap, if water and soap were observed at the handwashing place, and if use of water and soap was observed during the handwashing demonstration. This study will use the composite measure of handwashing with soap for all the bivariate and multivariate analyses, unless stated otherwise.

The exposure variables of interest include psychosocial and structural factors that may influence handwashing behaviors. A psychosocial factor examined in this study is people’s motivations for handwashing. Respondents were asked what motivates them to wash their hands in an open-ended question without any prompt or any limit to the number of responses that they can give. Each response was recorded as one of the nine reasons derived from literature search on sanitation and hygiene in Indonesia (1 = to prevent the spread of disease, 2 = to be clean, 3 = to smell good, 4 = to get rid of dirt/smell/sticky things on my hands, 5 = religious reasons/beliefs, 6 = was told it was the right thing to do, 7 = because that’s what everyone does, 8 = don’t know, 9 = other), and indicator variables (0 = not mentioned, 1 = mentioned) were constructed for each motivational factor.

This study also assessed seven handwashing opportunities or moments, which consist of before cooking, before eating, after eating, before feeding a child, after cleaning child stools, after defecation, and after work. As with motivations for handwashing, respondents were asked when they usually wash their hands without prompt, and indicator variables were made for each handwashing opportunity (0 = not mentioned, 1 = mentioned). For a structural factor, access to handwashing facilities in the household was directly observed and recorded with the following categories: within 10 paces of the toilet facility, within 10 paces of the kitchen, elsewhere in the household, outside of the yard, and no specific place.

Control variables for this study consist of respondents’ age, sex, education, district, household size, access to water and sanitation, and wealth quintile. As a proxy measure of socioeconomic status, the wealth quintile was developed with 12 variables that represent household assets and characteristics (i.e., radio, TV, mobile phone, telephone, refrigerator, motorcycle, bicycle, animal drawn cart, car/truck, boat with motor, own agriculture land, own farm animals) by conducting the principal component analysis [27].

This study employed STATA 13 to perform univariate, bivariate, and multivariate analyses [28]. Bivariate and multivariate analyses were adjusted for the complex survey design by using available sampling units. Descriptive statistics of all study variables were calculated to provide the general characteristics of the study population (Table 1). The proportion of respondents who wash hands with soap by study characteristics was also estimated while performing the test of independence. Bivariate associations between key exposure variables and the outcome variable were assessed with generalized linear models (GLM) with Poisson Family and Log Link to produce the prevalence ratio of hand washing with soap. Lastly, multivariate nested GLM models were run to estimate the extent to which socio-demographic characteristics, access to water and sanitation, and wealth quintiles moderate the main effect of each independent variable on handwashing with soap. Model 1 incorporated all of the control variables including respondents’ age, household size, education, biological sex, district, household wealth, and self-reported access to water and a private toilet as the baseline specification. Model 2 added people’s motivations for handwashing to the baseline model. Model 3 added directly observed handwashing locations in the household to the baseline specification. Model 4 included self-reported moments when respondents usually wash hands. Model 5 included all of the independent and control variables.

Due to the issue of multicollinearity (e.g., Variance Inflation Factor ≥5), Alor and Sumba Timur districts were both included as the reference group of the district variable in the multivariate analysis. This study also examined if the presence of a handwashing place near the kitchen and the toilet is associated with handwashing with soap before cooking and after defecation, respectively. Neither of the hypothesized interaction effects was statistically significant, and thus, multivariate models only assessed the main effects of independent variables.

## 3. Results

The descriptive characteristics of the study participants are summarized in Table 1. The majority of respondents were aged 35–55 years and came from a household with four to six people. Over 75% of respondents completed at least primary education. Male and female respondents were almost equally represented in this study. The sample distribution by districts ranged from 9.1% in Jayapura to 23.9% in Luwu Utara in accordance with the population size of each district. Each wealth quintile represented approximately 20% of respondents. Most of the households (87.9%) had access to water throughout the year, and 61.5% had a private sanitation facility in the household overall.

The top three self-reported reasons or motivations for handwashing were to be clean (89.6%), to prevent the spread of disease (52.9%), and to get rid of dirt/smell/sticky things (37.5%). Approximately 60% of households had access to a handwashing place within 10 paces of the toilet or the kitchen, and 19% had a handwashing station in other locations around the household. However, 21.3% did not have a specific location for handwashing. Lastly, almost 90% of respondents reported to wash hands before eating, with only 56.6% of people reporting to wash hands after eating and 52.1% after defecation. Only 7.6% and 13.4% of respondents reported to wash hands before feeding a child and after cleaning child stools, respectively. These results suggested that many respondents do not wash hands at all of the critical moments including before cooking, before eating, before feeding a child, after cleaning child stools, and after defecation.

Major differences were observed for the prevalence of handwashing with soap between the individual measures of handwashing and the composite measure (Table 2). While self-reports, direct observations of handwashing materials, and direct observations of handwashing demonstration suggested that over 70% of respondents wash hands with soap, the composite measures provide much lower estimates ranging from 55.7% to 63.9%. The highest estimate of handwashing with soap was 75.2% by direct observations of handwashing demonstration. Some respondents may have demonstrated a better handwashing behavior than their typical behavior, partially due to the presence of the observer. The lowest estimate for handwashing with soap was 55.7% by the composite measure of observed handwashing materials and handwashing demonstration. Adding a criterion of self-reported use of water and soap to this composite measure did not change the estimate of handwashing with soap.

### 3.1. Bivariate Analysis

The prevalence of handwashing with soap at critical moments was very low. Based on the composite measure, only 26.7% and 52.0% of respondents were estimated to wash hands with soap after defecation and before eating, respectively. The prevalence was even lower before feeding children (4.0%) and after cleaning child stools (6.9%), and only 1.8% of respondents were estimated to wash hands with soap at these four critical moments.

The proportion of older adults who washed hands with soap (49.4%) was lower than that of young adults (56.3%) and middle-aged adults (57.0%) (See Supplementary Materials, Table S1). The estimated prevalence of handwashing with soap by household size ranged from 51.9% in small households to 59.6% in large households. Female and male respondents had the same prevalence of handwashing with soap, at 56%. Statistical tests of independence suggested that age, household size, and sex were not significantly associated with the handwashing outcome in this study (Supplementary Materials, Table S1).

**Figure 1:**
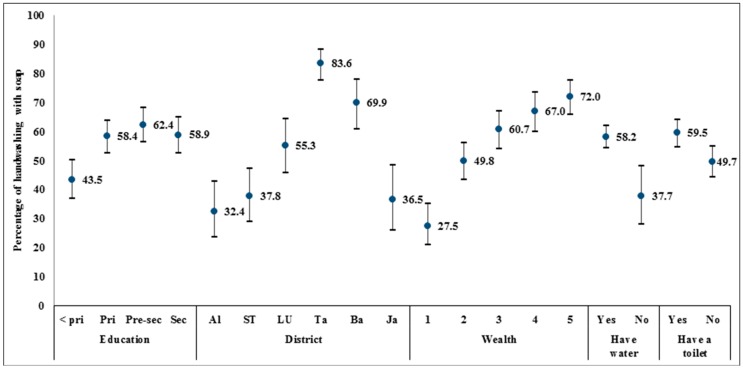
Percentage of respondents who washed hands with soap by education, district, wealth, and access to water and sanitation. Notes: <pri = Less than primary education, Pri = Primary education completed, Pre-sec = Pre-secondary education, Sec = Secondary education or higher. Al = Alor, ST = Sumba Timur, LU = Luwu Utara, Ta = Takalar, Ba = Barru, Ja = Jayapura. Wealth 1 = Poorest, 2 = Poorer, 3 = Middle, 4 = Richer, 5 = Richest.

The respondent’s education, the district the household was located in, household wealth, and access to water and sanitation were significantly associated with the behavior of handwashing with soap, and the calculated prevalence is presented in Figure 1. While over 58% of respondents with primary, pre-secondary, or secondary or higher education washed their hands with soap, only 43.5% of respondents with less than primary education washed their hands with soap. A large gap in the prevalence of handwashing with soap also existed between the six districts, ranging from 32.4% in Alor to 83.6% in Takalar. A positive curvilinear trend in the percentage of handwashing with soap was found for the quintiles of wealth. In the lowest wealth quintile, only 27.5% of respondents washed hands with soap, while 72.0% in the highest quintile performed handwashing with soap. Lastly, the majority of respondents, 58.2% and 59.5%, respectively, reported to have access to water and a private toilet washed their hands with soap.

Table 3 presents the proportion of respondents who wash hands with soap by self-reported motivations for handwashing, observed locations of the place for handwashing, and self-reported handwashing moments. The estimated prevalence of handwashing with soap by motivations ranged from 30.7% to 75.0%. Four motivations for handwashing—disease prevention, good smell, aesthetic purposes, and the belief that others in the community wash their hands with soap (i.e., descriptive norms)—were significantly associated with handwashing with soap, though not always there was a positive association. Having a motivation for good smell was associated with a 46.2% higher prevalence of handwashing with soap than that of respondents without this motivation. In contrast, motivations for disease prevention, removal of dirt, and the belief that others in the community wash their hands were associated with a lower prevalence of handwashing with soap than those who did not report these motivations.

The majority of respondents who had access to a handwashing place within 10 paces of the kitchen and the toilet facility was estimated to wash their hands with soap. Compared to respondents without a specific location for handwashing in the household, the prevalence of handwashing with soap was on average 24% and 37% higher among those respondents who had a handwashing place within 10 paces of the kitchen and the toilet, respectively.

The estimated prevalence of handwashing with soap by self-reported handwashing moments ranged from 39.3% before cooking to 63.0% after eating. Five handwashing moments, including before cooking, before eating, after eating, after defecation, and after work were significantly associated with handwashing with soap. The direction of associations, however, was not uniform. Reporting to wash hands before eating and after eating were associated with a higher prevalence of handwashing with soap than those who did not report to wash hands at these respective moments. The other handwashing moments (i.e., before cooking, after defecation, after work), however, were negatively associated.

### 3.2. Multivariate Analyses

The results of nested GLM regression analysis are presented in Table 4. In Model 1, household size, a private sanitation facility in the household, district, and wealth levels were significantly associated with handwashing with soap, controlling for other variables. The prevalence of handwashing with soap in the highest wealth quintile was 89% higher than that of the lowest quintile of wealth. These findings are in accordance with previous research that highlighted the roles of socio-economic and structural factors as potential determinants of handwashing with soap.

In Model 2, motivations of good smell and interpersonal influence were positively associated with handwashing with soap while negative associations were found for the aesthetic reason (i.e., to get rid of dirt, smell, sticky things on hands) and the descriptive norm (i.e., perceiving that other people wash hands with soap). By holding other variables constant, respondents who washed their hands for good smell and interpersonal influence (i.e., being told that it was a right thing to do) were associated with having 26.2% and 26.0% higher prevalence of handwashing with soap than other respondents without these motivations. Having a motivation to remove dirt, smell, or sticky materials on their hands was associated with 20.6% lower prevalence of handwashing with soap than those respondents without this motivation.

In Model 3, respondents who have a handwashing place within 10 paces of the kitchen and the toilet were associated with 28.2% and 46.7% higher prevalence of handwashing with soap, respectively, than those respondents who do not have a specific handwashing place. Thus, the presence of handwashing stations in the household may be a key structural determinant or enabling environment of handwashing with soap. By adding the handwashing place to the baseline specification, the association between self-reported ownership of a private toilet and handwashing with soap was no longer statistically significant. This result suggests that the presence of handwashing stations may mediate the independent effect of the sanitation variable on handwashing with soap, highlighting the need for promoting handwashing stations around the household sanitation facility or the kitchen to facilitate handwashing with soap.

In Model 4, four handwashing moments, including before cooking, before eating, after eating, and before feeding children, were significantly associated with handwashing with soap when holding other variables constant. The prevalence of handwashing with soap among respondents who reported to wash hands before cooking was 26.0% lower than that of respondents who did not report to wash hands at this handwashing moment. This finding suggests that people do not often use soap for their handwashing before cooking, and consequently food preparation may remain an important pathway for food contamination. Respondents who reported to wash their hands at the other three handwashing moments had a higher prevalence of handwashing than the respondents who did not report to wash hands at these moments. In this model, household size lost its significant association with handwashing with soap. This may be a reason that the independent effect of household size is significant in the previous regression models, which do not assess the association of handwashing moments with handwashing behaviors. The analysis suggests that in Model 4, self-reported handwashing moments may mediate the association between household size and handwashing with soap.

Most of the variables maintain their significant association with handwashing with soap in Model 5, except for a few self-reported motivations and handwashing moments. In this model, cleanliness, good smell, aesthetic motivations, religious reasons, and interpersonal influence have significant associations with handwashing with soap while the descriptive norm is no longer significant. Self-reported handwashing moments that include before cooking, after eating, and after cleaning child stools were significantly associated with the outcome.

## 4. Discussion

This study explored potential determinants of handwashing with soap in six districts across three provinces of Indonesia. The results suggested that structural factors, namely household wealth levels, district of residence, and presence of handwashing infrastructure within 10 paces of the kitchen and the toilet, are associated with greater handwashing with soap in the context of this study. Motivations for handwashing, such as good smell and interpersonal influence, were also identified as factors associated with handwashing with soap. The analysis further highlighted that handwashing with soap is more common at certain handwashing moments, mainly after eating and after cleaning child stools, than other critical times, such as after defecation or before food preparation.

The estimated prevalence of handwashing with soap by the composite measure was lower than that of individual measures of handwashing in this study. Although this study cannot assess the validity of the composite measure by comparing the estimated prevalence with that of structured observations, a more conservative estimate of handwashing behavior was obtained. The findings in this study can be interpreted to indicate that the combination of multiple indicators, rather than one indicator, may be more useful for household surveys and reduce the risk of overestimating handwashing behaviors. The Demographic and Health Surveys (DHS) and the Multiple Indicator Cluster Survey (MICS) only collect data on the presence of soap and water for handwashing in the household as the proxy measure of handwashing behaviors. Handwashing measurement in these large household surveys could potentially be strengthened by including the direct observation of handwashing demonstration and developing a composite measure to estimate the prevalence of handwashing with soap in low- and middle-income countries.

Descriptive analysis revealed that Alor district has the lowest prevalence of handwashing with soap, which could have been induced by limited access to water in this district. A further analysis of the data found that only 46.2% of households in Alor were estimated to have access to water throughout the year, compared to at least 87% or higher in other districts. In focus group discussions conducted by TNS, a female participant from Alor also stated that “I will not let my kids wash their hands unless we have enough water, since getting water is difficult and our priority is for drinking and cooking” [11]. Adequate access to drinking water is, therefore, an important precursor to perform handwashing with soap. Nonetheless, a multivariate analysis did not find a significant association between having year-round access to water and handwashing with soap. Future research may modify this indicator and collect information on the quantity of water that households can access.

The drivers that form hygiene habits or change individual and community-level hygiene behaviors are not fully understood. In this study, disease prevention did not appear to be a primary driver for handwashing with soap. By recognizing that handwashing may not be always facilitated by health-related motivations, previous research has explored the potential roles of emotional drivers (e.g., disgust, comfort) and sociocultural factors (e.g., norms, habit formation) for handwashing promotion [16,19]. Disgust has also been used to promote handwashing with soap in different cultural contexts [29,30], and a recent cluster-randomized trial revealed that handwashing behaviors can be affected by addressing disgust as a key emotional driver [31]. In this study, a motivation for handwashing—removal of dirt, smell, or sticky things on the hands—was used as a proxy measure for disgust. This motivation, however, was found to be associated with reduced levels of handwashing with soap. This finding could suggest that this was a poor proxy of disgust or that disgust was not a compelling driver of increased handwashing. Additional evidence is needed to understand how emotional drivers may more effectively impact handwashing with soap.

Interpersonal influence was also identified as an influential factor for handwashing promotion. A qualitative study with the same study population revealed that local authorities, community health workers, and religious leaders are influential sources of information on sanitation and hygiene in this study context [11]. Accordingly, promoting handwashing with soap through these information providers may effectively enhance people’s handwashing behaviors. Moreover, evidence suggests that habits can play a major role in shaping people’s handwashing behaviors [16,32]. Government-led handwashing promotion efforts for adult populations may be complemented with school-based handwashing interventions to form the habit of handwashing with soap among children though the experience of UNICEF Indonesia has been that handwashing rates in schools still remain low, and this message needs constant reinforcement for sustainability.

Handwashing moments appeared to provide varying levels of physical cues to induce handwashing with soap. The results suggested that handwashing with soap was more commonly practiced after eating and less commonly practiced before cooking. While the exact reasons for limited handwashing with soap before cooking remain unclear in this study, people may be washing hands with soap inconsistently or wash hands with water only. This finding is in accordance with earlier formative research in Serang district of Indonesia, which reported that new mothers infrequently and inconsistently practiced handwashing at critical moments, including before cooking [25]. While hygiene interventions for both men and women remain vital for improving public health, tailoring educational messages to a specific population group or sex may improve handwashing practices before cooking. Additionally, previous research identified after eating as a common handwashing moment despite the low priority of this practice from a public health perspective [16]. The cultural practice of using hands to eat food may be a driver for people to wash hands with soap after completing their meal. Lastly, the low level of handwashing with soap after defecation identified in this study remains a major public health concern in Indonesia where open defecation is commonly practiced [8]. To protect the population from infectious diseases, the importance of handwashing with soap and ownership of household sanitation facilities needs to be further emphasized and promoted in this context.

There are some important limitations to this study. First, self-reported data could be influenced by social desirability bias [20]. The study team informed potential study participants that the purpose of this study was to ask them about water, sanitation, and hygiene (WASH) and health conditions in the household. This process could have prompted respondents to mention disease prevention as a motivator for handwashing practice and report handwashing moments that people should wash hands rather than their true hygiene behaviors. To minimize this bias, however, this study explored respondents’ handwashing motivations and handwashing moments through an open-ended question without the use of any prompts. Second, this study employed cross-sectional data for statistical analyses, so the temporality of independent and dependent variables cannot be ascertained. Reverse causation could be a possibility. Third, handwashing with soap estimated by the composite measure does not necessarily reflect people’s true behavior. A great deal of empirical evidence has confirmed that self-reported handwashing behaviors tend to be overestimated and are not reliable, and structured observations may be subject to reactivity [20]. As previously stated, other measurement methods also have both advantages and disadvantages of measuring handwashing behaviors. As such, this study combined three measurement methods to estimate the prevalence of handwashing with soap. While the estimated prevalence of handwashing with soap was 71.2% by only using the self-reported measure, it was estimated as 55.7% based on the composite measure. Thus, this study addressed the measurement errors to the extent that is possible with the available data. Lastly, data on the primary sampling unit were not available in this study, so the tertiary sampling unit was used to adjust for the clustering effect as the best available option. Although the point estimate would not be altered, confidence intervals and statistical significance are likely to be different if the primary sampling unit data were available.

To generate a research agenda for the future, we conducted a post hoc correlation analysis to assess the association between self-reported motivations for handwashing and three handwashing moments—after defecation, before eating, and after cleaning child stools. This analysis revealed that motivations for disease prevention, cleanliness, good smell, interpersonal influence, and descriptive norms were positively associated with self-reported handwashing after defecation. Cleanliness and good smell were positively associated with handwashing before eating while a negative association was found for the aesthetic motivation. Lastly, disease prevention and aesthetic motivations were positively associated with handwashing after cleaning child stools. Accordingly, motivational drivers differently function to influence people’s handwashing practices at each critical handwashing moment. To promote handwashing with soap at critical moments more effectively, future research can further examine what motivations are associated with people’s handwashing practices at each handwashing moment.

## 5. Conclusions

This study identified some of the potential determinants of handwashing with soap by highlighting the substantial effects of structural and psychosocial factors. Physical proximity to handwashing stations has been shown to facilitate handwashing behaviors. Future interventions that send the message of good smell and comfort associated with handwashing may be also effective to promote the practice of handwashing with soap. More efforts are necessary to understand which key drivers for handwashing could effectively increase the practice of handwashing with soap at each critical moment, along with increased operational guidance on how to change his personal hygiene behavior at scale and maintain the behavior as a habit.

Handwashing with soap is a cost-effective strategy to reduce the global burden of disease, yet this hygiene behavior has not been fully recognized as a global health priority. While water and sanitation have been included in the Millennium Development Goals (MDGs) and the Sustainable Development Goals (SDGs), the progress on hygiene promotion has not been sufficiently tracked globally. While the evidence base of handwashing measurement is not complete, this study revealed a potential utility of composite measures to monitor and evaluate the progress of handwashing promotion. Developing credible indicators to monitor handwashing behaviors can be the first step to further highlight the importance of hygiene behaviors in global health.

## Figures and Tables

**Table 1:** Descriptive analysis of study variables, *n* = 1696.

Characteristics	*n* (%)
Age	
Young (aged 18–35)	610 (36.0)
Middle (aged 36–55)	848 (50.0)
Older (aged 56 or older)	238 (14.1)
Household size	
Small (1–3 people)	461 (27.2)
Middle (4–6 people)	963 (56.8)
Large (7 or more people)	271 (16.0)
Education	
Less than primary education	418 (24.6)
Primary education	426 (25.1)
Pre-secondary education	346 (20.4)
Secondary or higher	506 (29.8)
Sex	
Female	853 (50.3)
Male	843 (49.7)
District	
Alor	245 (14.4)
Sumba Timur	278 (16.4)
Luwu Utara	404 (23.9)
Takalar	364 (21.5)
Barru	251 (14.8)
Jayapura	154 (9.1)
Wealth quintile	
Poorest	333 (19.6)
Poorer	327 (19.3)
Middle	336 (19.8)
Richer	364 (21.4)
Richest	337 (19.9)
Reported to have water for household needs throughout the year	1492 (87.9)
Reported to have a private toilet	1043 (61.5)
Self-reported reasons for hand washing	
To prevent the spread of disease	896 (52.9)
To be clean	1519 (89.6)
To smell good	311 (18.3)
To get rid of dirt/smell/sticky things	636 (37.5)
Religious reasons	11 (0.7)
Being told that it was the right thing to do	65 (3.8)
Because that is what everyone does	26 (1.5)
Observed place for habitual hand washing	
No specific place	361 (21.3)
Outside of yard	95 (5.6)
Elsewhere in home or yard	228 (13.4)
Within 10 paces of the kitchen/cooking space	746 (44.0)
Within 10 paces of the toilet facility	266 (15.7)
Self-reported moments when respondents usually wash hands	
Before cooking	270 (15.9)
Before eating	1520 (89.7)
After eating	926 (54.6)
Before feeding a child	129 (7.6)
After cleaning feces of a child	227 (13.4)
After defecation	884 (52.1)
After work	749 (44.2)

**Table 2:** Handwashing (HW) with soap estimated by self-reports, direct observations, and a composite measure, *n* = 1696.

Measurement Methods	*n* (%)
Self-reported use of water and soap for handwashing (HW1)	1208 (71.2)
Observed presence of water and soap at the handwashing place (HW2)	1190 (70.2)
Observed use of water and soap during handwashing demonstration (HW3)	1275 (75.2)
HW1 and HW2	995 (58.7)
HW1 and HW3	1084 (63.9)
HW2 and HW3	944 (55.7)
HW1 and HW2 and HW3 (Outcome variable of this study)	944 (55.7)
HW1 and HW2 and HW3 and reported to wash hands after defecation	453 (26.7)
HW1 and HW2 and HW3 and reported to wash hands before eating	882 (52.0)
HW1 and HW2 and HW3 and reported to wash hands before feeding children	67 (4.0)
HW1 and HW2 and HW3 and reported to wash hands after cleaning child stools	117 (6.9)
HW1 and HW2 and HW3 and reported to wash hands at these four moments	31 (1.8)

**Table 3:** Unadjusted generalized linear models (GLM) regression (Family: Poisson, Link: Log) of handwashing with soap by selected variables, *n* = 1696.

	Number and Percentage of Respondents with This Response Who Wash Hands with Soap *n* (%)	Number and Percentage of Respondents without This Response Who Wash Hands with Soap *n* (%)	PR (95% CI)	*p*-Value
Self-reported motivations for handwashing				
To prevent the spread of disease	447 (49.8)	498 (62.2)	0.80 (0.72, 0.89)	<0.001
To be clean	833 (54.8)	112 (63.2)	0.87 (0.75, 1.01)	0.060
To smell good	233 (75.0)	711 (51.3)	1.46 (1.31, 1.63)	<0.001
To get rid of dirt/smell/sticky things	288 (45.4)	656 (61.9)	0.73 (0.65, 0.83)	<0.001
Religious reasons	7 (59.6)	938 (55.7)	1.07 (0.65, 1.76)	0.786
Being told that it was a right thing to do	38 (58.7)	906 (55.6)	1.06 (0.85, 1.31)	0.612
Because that is what everyone does	8 (30.7)	936 (56.1)	0.55 (0.31, 0.98)	0.043
Directly observed handwashing place				
Outside of the yard	33 (34.3)	174 (48.2)	0.71 (0.49, 1.04)	0.077
Elsewhere in the household	116 (50.8)	174 (48.2)	1.05 (0.85, 1.30)	0.627
Within 10 paces of the kitchen/cooking space	445 (59.7)	174 (48.2)	1.24 (1.03, 1.49)	0.024
Within 10 paces of the toilet facility	177 (66.3)	174 (48.2)	1.37 (1.12, 1.69)	0.003
Self-reported moments of habitual handwashing				
Before cooking	106 (39.3)	838 (58.8)	0.67 (0.56, 0.80)	<0.001
Before eating	882 (58.0)	62 (35.5)	1.63 (1.27, 2.09)	<0.001
After eating	584 (63.0)	360 (46.8)	1.35 (1.18, 1.53)	<0.001
Before feeding a child	67 (52.2)	877 (56.0)	0.93 (0.76, 1.14)	0.496
After cleaning feces of a child	117 (51.6)	827 (56.3)	0.92 (0.79, 1.06)	0.243
After defecation	453 (51.3)	491 (60.5)	0.85 (0.76, 0.94)	0.002
After work	349 (46.5)	596 (62.9)	0.74 (0.65, 0.84)	<0.001

PR = prevalence ratio. Notes: Respondents were free to mention multiple motivations for handwashing and moments when they usually wash hands. Each self-reported motivation is a binary variable in which respondents who did not mention a given motivation represent a reference group.

**Table 4:** Multivariate GLM regression (Family: Poisson, Link: Log) of handwashing with soap presented in prevalence ratio. Indonesia, 2014, *n* = 1696.

	Model 1	Model 2	Model 3	Model 4	Model 5
Respondents’ age (Ref: 18–35 years)					
36–55	1.022	1.031	1.031	1.052	1.069
56 or older	0.926	0.910	0.937	0.946	0.942
Household size (Ref: 1–3 people)					
4–6	1.031	1.052	1.036	1.008	1.033
7 or more	1.180 *	1.190 *	1.146 *	1.112	1.102
Education (Ref: Less than primary)					
Primary	0.943	0.924	0.966	0.932	0.947
Pre-secondary	0.936	0.912	0.974	0.932	0.953
Secondary or higher	0.961	0.948	0.983	0.943	0.956
Female (Ref: Male)	1.002	0.997	0.981	0.985	0.969
District (Ref: Alor and Sumba Timur)					
Luwu Utara	1.141	1.211	1.149	1.032	1.091
Takalar	1.788 ***	1.785 ***	1.663 ***	1.755 ***	1.626 ***
Barru	1.482 **	1.532 ***	1.443 **	1.479 **	1.481 **
Jayapura	0.764	0.732	0.654 *	0.719	0.605 **
Wealth Quintile (Ref: Poorest)					
Poorer	1.535 **	1.465 **	1.587 ***	1.420 **	1.438 **
Middle	1.666 ***	1.586 **	1.683 ***	1.567 **	1.538 **
Richer	1.737 ***	1.706 ***	1.757 ***	1.626 ***	1.654 ***
Richest	1.876 ***	1.780 ***	1.833 ***	1.774 ***	1.691 ***
Have access to water (Ref: No)	1.069	1.060	1.133	1.028	1.089
Have a private sanitation facility (Ref: No)	1.135 *	1.120 *	1.093	1.115	1.081
Self-reported motivations for handwashing:					
To prevent the spread of disease		1.087			1.078
To be clean		0.890			0.871 *
To smell good		1.262 ***			1.234 ***
To get rid of dirt/smell/sticky things on hands		0.794 ***			0.819 ***
Religious reasons		1.420			1.574 *
Being told that it was the right thing to do		1.260 *			1.230 *
Because that is what everyone does		0.571 *			0.605
Observed HW place (Ref: No specific place)					
Outside of yard			0.878		0.836
Elsewhere in the household			1.086		1.096
Within 10 paces of the kitchen/cooking space			1.282 **		1.267 **
Within 10 paces of the toilet facility			1.466 ***		1.362 ***
Self-reported handwashing moments:					
Before cooking				0.740 ***	0.763 ***
Before eating				1.271 *	1.223
After eating				1.177 **	1.163 **
Before feeding				1.271 *	1.182
After cleaning child stools				1.119	1.192 *
After defecation				1.022	1.007
After work				0.918	0.944
Adjusted Wald test: F-statistic	7.02	7.59	7.35	7.55	6.67
Df	(18, 327)	(25, 320)	(22, 323)	(25, 320)	(36, 309)

Notes: * *p* < 0.05, ** *p* < 0.01, *** *p* < 0.001. Respondents were free to mention multiple motivations and handwashing moments in an open ended question without any prompt. Df = Degrees of freedom.

## Supplementary Materials

The following are available online at www.mdpi.com/1660-4601/13/9/868/s1, Table S1: Proportion of respondents who wash hands with soap by study variables.
