# Supplementary material for: Exploring Determinants of Handwashing with Soap in Indonesia: A Quantitative Analysis
Source: Int J Environ Res Public Health. 2016 Sep 1;13(9):868. doi: 10.3390/ijerph13090868 (PMC5036701; doi:10.3390/ijerph13090868)
Supplement: Supplementary file 1 [file ijerph-13-00868-s001.pdf]

Supplementary Materials: Exploring Determinants of Handwashing with Soap in Indonesia: A Quantitative Analysis

Mitsuaki Hirai, Jay P. Graham, Kay D. Mattson, Andrea Kelsey, Supriya Mukherji and Aidan A. Cronin

Table S1. Proportion of respondents who wash hands with soap by study variables, $n = 1696$.

	% HWWS (95% CI)	*p*-Value
Age		0.13
Young (18–35 years)	56.3 (51.0, 61.5)	
Middle (36–55 years)	57.0 (52.6, 61.3)	
Older (56 years or older)	49.4 (42.4, 56.4)	
Household size		0.16
Small (1–3 people)	51.9 (45.6, 58.0)	
Middle (4–6 people)	56.4 (52.1, 60.6)	
Large (7 or more people)	59.6 (52.8, 66.1)	
Education		**0.0001**
Less than primary	43.5 (36.9, 50.3)	
Primary	58.4 (52.6, 63.9)	
Pre-secondary	62.4 (56.3, 68.1)	
Secondary or higher	58.9 (52.6, 64.9)	
Sex		0.96
Female	55.7 (51.6, 59.8)	
Male	55.6 (51.0, 60.2)	
District		**<0.0001**
Alor	32.4 (23.6, 42.7)	
Sumba Timur	37.8 (29.1, 47.4)	
Luwu Utara	55.3 (45.8, 64.5)	
Takalar	83.6 (77.6, 88.2)	
Barru	69.9 (60.7, 77.8)	
Jayapura	36.5 (26.1, 48.4)	
Wealth Quintile		**<0.0001**
Poorest	27.5 (21.0, 35.1)	
Poorer	49.8 (43.4, 56.2)	
Middle	60.7 (54.0, 67.0)	
Richer	67.0 (59.8, 73.5)	
Richest	72.0 (65.8, 77.5)	
Have water for household needs throughout the year		**0.0003**
Yes	58.2 (54.2, 62.0)	
No	37.7 (28.1, 48.2)	
Have a private toilet		**0.0049**
Yes	59.5 (54.7, 64.0)	
No	49.7 (44.3, 55.0)	

Notes: HWWS = Handwashing with soap. A chi-square test was used for this analysis, and bold values are statistically significant ($p < 0.05$).
